# Attributes of screening and vaccination for cervical cancer: insights of an online survey among female school teachers of Kota, Rajasthan, India

**DOI:** 10.34172/hpp.2021.07

**Published:** 2021-02-07

**Authors:** Aparajita Sharma, Bijit Biswas, Bhawna Sati

**Affiliations:** ^1^Department of Public Health, Jodhpur School of Public Health, Maulana Azad University, Jodhpur, Rajasthan, India; ^2^Department of Community and Family Medicine, All India Institute of Medical Sciences, Patna, Bihar, India

**Keywords:** Uterine cervical neoplasms, School teachers, Knowledge, Attitude, Early detection of cancer, Vaccination

## Abstract

**Background:** Cervical cancer is largely preventable. The study was aimed to find out the attributes of screening and vaccination for cervical cancer among female school teachers of Kota, Rajasthan, India.

**Methods:** It was an observational study, cross-sectional in design conducted among female school teachers of Kota, Rajasthan, India using a predesigned structured questionnaire administered by Google Form during the months of March to May, 2020. In total, 397 schoolteachers from 18 different schools of Kota participated in our survey.

**Results:** Among the study subjects, 33 (8.3%) had ever undergone screening for cervical cancer, whereas only 12 (3.0%) had taken vaccine against it. In multivariable logistic regression analysis; age (>40 years) [adjusted odds ratio (AOR): 5.7 (2.0-15.8)], correct knowledge regarding frequency of screening (yes) [AOR: 6.0 (2.4-15.5)], positive attitude for the disease (yes) [AOR:3.0 (1.1-8.0)] and gynaecologist consultation behaviour (periodic) [AOR: 1.4 (1.1-8.6)] were significant attributes of ever undergoing screening for cervical cancer. On the other hand, themultivariable significant attributes of vaccination were age (≤40 years) [AOR: 7.6 (1.5-38.9)]and positive attitude for the disease (yes) [AOR: 6.4 (1.1-38.2)].

**Conclusion:** Acceptance of screening and vaccination for cervical cancer among the study subjects was found to be quite low. Policymakers should more actively involve school teachers in generating awareness and in raising the demand for cervical cancer screening and vaccination in their native communities to curb the disease in the country.

## Introduction

Cervical cancer is the fourth most common cancer in women after breast, colorectal and lung cancer worldwide with half a million new cases and about three hundred thousand deaths annually.^1,2^ Majority of these deaths are attributed to cervical cancer especially in lower- and middle-income countries. In India, about a hundred thousand women are diagnosed with cervical cancer every year, half of which succumb to the disease. Moreover, the country contributes about one-fifth of global annual cervical cancer incidence.^1,3^

Cervical cancer is largely preventable. In the primary level of prevention, human papillomavirus (HPV) vaccine may be provided which protects its recipient from HPV infection. HPV is associated with 70% of cervical cancer cases.^4-6^ Efficacy and cost-effectiveness of HPV vaccine both declines with increasing age and sexual exposure. Therefore, it is seldomly recommended after the age of 26.^4,6-8^ In the secondary level, periodic cervical cancer screening helps in early diagnosis and treatment of the ailment.^9^ Pap test is the most widely used cervical cancer screening method.^10^ In India, cervical cancer screening is routinely done at 30-65 years aged women in every 5 years.^11^ However, the American Cancer Society (ACS) recommends initiation of cervical cancer screening at 21 years and to be repeated in every 3 years.^12^

As per the fourth round of the National Family Health Survey (NFHS-4), only 22.3% of women aged 15-49 years in India had ever undergone cervical cancer screening in their life.^13^ In Rajasthan, this was even lower (18.9%).^14^ The data reported by NFHS-4 related to cervical cancer screening reflected the need of strategies and interventions to improve cervical cancer screening and vaccination related practices in women of the country. Female school teachers represent educated and working-class women of the community. They are potential percolators of awareness related to the disease in their native communities either directly by organising awareness generation community meetings or through their students. They can motivate their students and community dwelling women concerning cervical cancer screening and vaccination by oping these for themselves.^15^ To do all these, the attributes of their own screening and vaccination status for cervical cancer needs to be explored first. Although several prior foreign studies^16-20^ were conducted in this aspect but in Indian context, we could not find any prior evidences. On the other hand, NFHS reports prevalence of cervical cancer screening among women in the country but it does not provide any data on their vaccination status for the disease. Thus, this study was planned to find out the attributes of screening and vaccination for cervical cancer among female school teachers of Kota, Rajasthan, India.

## Materials and Methods

It was an observational study, cross-sectional in design conducted among 397 female school teachers of Kota district of Rajasthan, India during March to May, 2020. Kota district is situated in eastern Rajasthan. For administrative purposes, the schools in Kota district are divided into six school blocks. From the existing school block line list, Kota block was selected randomly without substitute. Further, the schools in Kota block are divided into 90 clusters. Among these line-listed school clusters, 18 (20%) were randomly selected without substitute. Then one school from each selected school cluster line-list (average cluster size: 10.6~11 schools per cluster) was selected randomly with substitute. For all random selection, simple random sampling method was used.^16^ In the absence of any comparable prior study in the study area, assuming that at least 18.9% (~19.0%) (as per NFHS-4)^14^ of the subjects in the study population have ever undergone screening for cervical cancer, 7% absolute precision at 95% confidence, design effect of 2 and response rate of 70%, the final minimum sample size was calculated to be 345. The sample size was calculated using ‘statulator’ which is an online sample size calculator.

The heads of all these selected 18 schools were contacted and explained regarding the purpose of the study. This was followed by their consent for participation. Then a line-list of all female school teachers working in these schools along with their email addresses and WhatsApp numbers was prepared. The selected schools had in total 505 in service female school teachers at the time of selection. During the study period, all of them were invited to participate in the survey through email and WhatsApp. Additionally, the principal investigator had given repeated telephonic reminders to the study subjects for participation. Finally, 397 of them had given consent and participated in the study (response rate 78.6%). Lack of digital literacy, disease awareness and moreover reluctance for participation might have affected the study enrolment.

The data was collected using a structured schedule which was developed based on the available literature. The schedule was initially pretested among 30 female school teachers of a different school (other than the 18 study schools) and modified accordingly. This pretested data was not included in the final analysis. Each item in the tool was initiated in English language followed by its Hindi (local language) description to increase its comprehensibility among the study participants. The schedule was administered using ‘Google Form’. It is a tool by Google Limited Liability Company (LLC) that allows collecting information from its users through a personalized survey or quiz. The collected information gets automatically synced to a connected dynamic Google excel sheet. The finally designed Google Form had seven sections in total which began with a short description regarding the purpose of the study, followed by the question “do you voluntarily agree to participate in the study” with options agree / disagree. The further items of the questionnaire were only administered if the participant agreed to participate in the study. If the participant disagreed to participate, the survey ended there itself. After obtaining the consent in the second section, study participants were asked, “Are you suffering from or have ever suffered from cervical cancer?” with options yes / no. If they answered ‘yes’, the survey ended there itself. If they answered ‘no’ the survey continued. The third section comprised of socio-demographic variables. The fourth section contained 31 knowledge items related to cervical cancer (K1-K31). The knowledge items had shown good internal consistency (Cronbach’s alpha: 0.871). This was followed by six attitude items in fifth section [A1-A6]. The attitude items had shown acceptable internal consistency (Cronbach’s alpha: 0.738). In the sixth section study participants were asked regarding their various practices related to the disease (gynaecologist consultation seeking behaviour; cervical cancer screening and vaccination status). In the final section study participants were thanked for being a part of the survey.

Some operational definitions used in the study were as follows :

*Family history of cancer:* To elicit this variable, study participants were asked whether any first degree relative of theirs was suffering or had suffered from any cancer. If the answer was yes, they were considered as having a family history of cancer.

*Adequate knowledge regarding the risk factors:*Those who had knowledge of at least eight risk factors (more than or equal to median risk factors known to the study subjects) of the disease were considered as having adequate knowledge regarding the risk factors.

*Adequate knowledge regarding the signs or symptoms:*Those who could name six signs or symptoms (more than median signs or symptoms known to the study subjects) of the disease were considered as having adequate knowledge regarding the signs or symptoms.

*Knew ideal age for initiation of screening:*Those who reported it to be either ‘at 21 years’ or ‘at 30 years’ were considered as having knowledge about ideal age of initiation of screening.^11,12^

*Correct knowledge regarding frequency of screening:* Those who opined it to be either ‘every three years’ or ‘every five years’ were considered to have correct knowledge regarding frequency of screening.^11,12^

*Knew ideal age of vaccination:*Those who reported it to be ‘9-14 years’ were considered as having correct knowledge regarding ideal age of vaccination.^7^

*Attitude score:* Those who agreed to first, second, fourth and fifth attitude items received ‘1’ rating for each. For the third attitude item, those who disagreed earned ‘1’ score for it. All other responses received ‘0’ score. All the attitude related items were summed to obtain the total attitude score.

*Positive attitude:* Those who have scored more than the median attained attitude score of 5 were considered as having a positive attitude regarding the disease.

*Periodic gynaecologist consultation:*Those who have opined to consult a gynaecologist either in ‘every year’ or ‘every three years’ were considered as seeking periodic gynaecologist consultation.

### Statistical analysis

Data were analysed using SPSS Inc. (Chicago, USA) (version 16). Data were expressed as median (IQR) for quantitative variables and frequency (percentage) for qualitative variables. At first, bivariate analysis was performed using the chi-square test. This was done to find out significant attributes of cervical cancer screening and vaccination among the study subjects. Then bivariate logistic regression analysis was done to find out the strength of the association between screening and vaccination with their various attributes. Finally, statistically associated variables in bivariate analysis were entered into the multivariable logistic regression model. This was done to find out multivariable attributes affecting cervical cancer screening and vaccination status among the study subjects. The strength of association between screening and vaccination with their various attributes was expressed as odds ratio (OR). The minimum acceptable confidence level and the maximum acceptable significance level for all statistics was set at 0.05.

## Results

**Figure 1:**
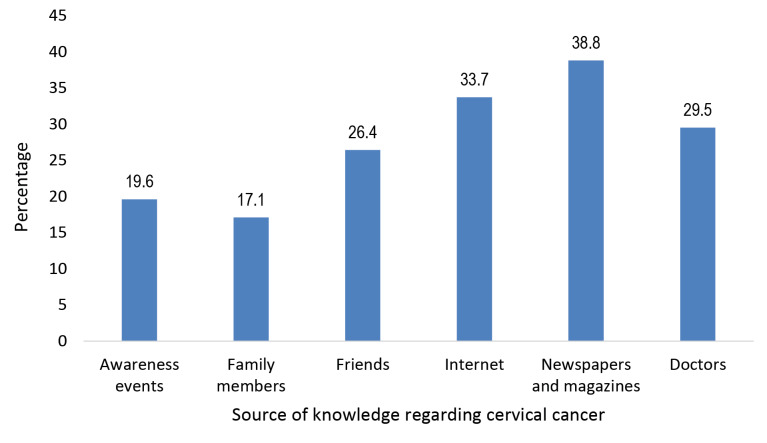
Bar chart showing distribution of the school teachers as per their source of knowledge regarding cervical cancer (multiple response) (n=397)

The median age of the study subjects was 40 years with interquartile range (IQR) of 34-49 years (range: 20-65 years). Majority of them were Hindu by religion (88.7%) followed by Muslim (4.0%), Sikh (3.0%) and Christian (2.8%). Considering ethnicity, about one-tenth (10.6%) of them belonged to other backward caste (OBC) while 5.0% and 4.5% of them were scheduled caste (SC) and scheduled tribe (ST) respectively. More than four-fifth of them were currently married (85.1%) while 2.8% and 2.5% of them were widowed and divorced respectively. The median per capita monthly family income (PCMI) was 204 USD with IQR of (118-340 USD) (range: 27-2044 USD). Half of them (51.9%) knew at least eight risk factors of the disease with median (IQR) of 8 (6-10) (range: 0-14) while about two-fifth of them (43.8%) had knowledge regarding at least six signs or symptoms of the disease with median (IQR) of 5 (3-7) (range: 0-10). The responses of different knowledge items and source of knowledge regarding the disease is depicted in Table 1 and Figure 1 respectively. About one-fourth of the study subjects (28.7%) had positive attitude regarding the disease with attitude score median (IQR) of 5 (4-6) (range: 1-6). Responses of the different attitude items of the study subjects are depicted in Table 2. Considering practice related to gynaecologist consultation, majority of them (86.6%) preferred it to be as per need while for 9.1% and 4.3% it was once in a year and three years respectively.

**Figure 2:**
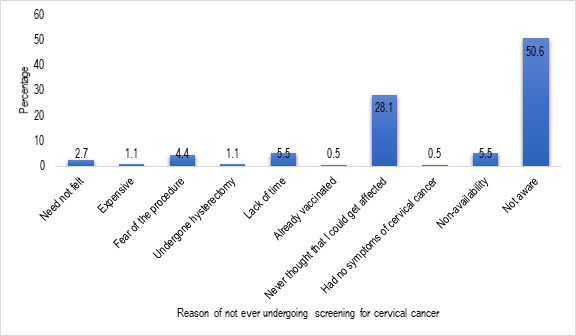
Bar chart showing the predominant reason for not ever undergoing screening for cervical cancer (n=364)

**Figure 3:**
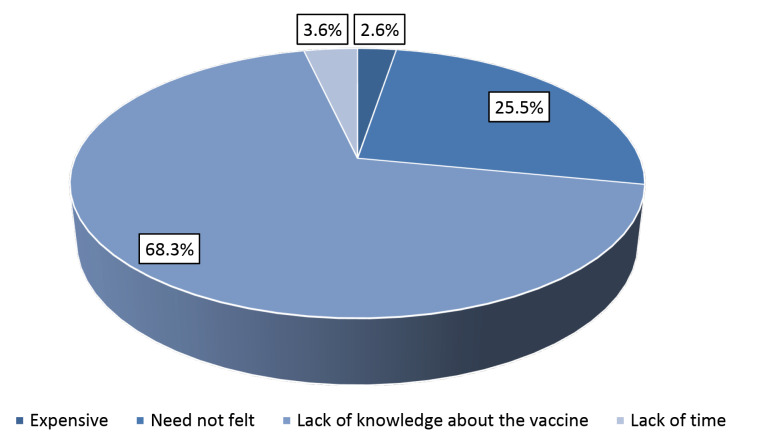
Pie chart showing the predominant reason for not taking vaccine against cervical cancer (n=385)

Among the study subjects, 33 (8.3%) had ever undergone screening for cervical cancer. Notably all of them had undergone screening for the disease by Pap test. The median age at the time of the last screening was 40 years with IQR of 35-48 years (range: 31-60 years). Frequency of Pap test till date for most was two (33.3%) with median (IQR) of 2 (1-3) (range: 1-5). The predominant reason for not undergoing cervical cancer screening till date is depicted in Figure 2. The screening acceptance rate was highest among Sikhs (33.3%) followed by Christians (27.3%) and Hindus (6.8%). Notably none of the Muslim study subjects had ever undergone screening. Only a few of the total participants, 12 (3.0%) had received vaccination for the disease with a median (IQR) age at vaccination of 30 (27-32) years (range: 25-34 years). The predominant reason for not taking vaccination for the disease is depicted in Figure 3. All the vaccine recipients were Hindu by religion.

Age, religion, ethnicity, knowledge regarding signs or symptoms, frequency of screening, vaccine availability, ideal age of vaccination, positive attitude and gynaecologist consultation behaviour were significant bivariate attributes of ever undergoing screening for cervical cancer. On the other hand; age, PCMI, knowledge of screening methods, ideal age of vaccination, positive attitude and gynaecologist consultation behaviour were the bivariate attributes affecting vaccination against the disease. In multivariable logistic regression analysis; age (>40 years) [adjusted odds ratio (AOR): 5.7 (2.0-15.8)], correct knowledge regarding frequency of screening (yes) [AOR: 6.0 (2.4-15.5)], positive attitude for the disease (yes) [AOR: 3.0 (1.1-8.0)] and gynaecologist consultation behaviour (periodic) [AOR: 1.4 (1.1-8.6)] were significant attributes of ever undergoing screening for cervical cancer. On the other hand, the multivariable significant attributes of vaccination were age (≤40 years) [AOR: 7.6 (1.5-38.9)] and positive attitude for the disease (yes) [AOR: 6.4 (1.1-38.2)] (Tables 3 and 4).

## Discussion

Only a few (8.3%) of the study participants had ever undergone a cervical cancer screening in our study which was quite low in comparison to NFHS-4 report for the state of Rajasthan (18.9%).^14^ The reason of the differences could be differences in study area (NFHS generates its data from different clusters of Rajasthan whereas we conducted our study in only a single school block of the state), study population age (NFHS reports data of 15-49 years aged women while our study was conducted among women aged 20-65 years), educational level (NFHS conducts its survey on all women irrespective of their educational status whereas our survey was limited to female school teachers only) etc. Considering studies conducted in other countries, Emmanuel et al^17^ (17.6%) and Abdullah et al^18^(38.0%) reported it to be higher compared to us. The variability of finding may be attributed to socio-cultural differences between the study population of those studies and ours.

In the present study, higher age emerged as a significant attribute of ever undergoing cervical cancer screening. This was unlike the observations of a Malaysian study^18^ which reported lower age as facilitator of cervical cancer screening. The reason for the differences could be low response rate in the Malaysian study. Thus, it might be possible that those who underwent or really wished to undergo screening for the disease only had participated in that study. Moreover, in India cervical cancer screening is recommended for women aged between 30 to 65 years (at a relatively higher age). Indian women seldomly seek healthcare for their sexual and reproductive health (SRH) before marriage unless medically indicated. All these might have reflected and influenced our study findings. Knowledge regarding frequency of screening and positive attitude significantly influenced cervical cancer screening status of our study subjects. This was in concordance with the findings of Emmanuel et al^17^ which reported both knowledge and attitude, whereas Ling et al^19^ and Ijezie & Johnson.^20^ which found only knowledge as attributes of ever undergoing screening for cervical cancer. It was an obvious finding as knowledge and attitude for a disease are known influencers of practice associated with it. Concerning periodic gynaecologist consultation, a study in Iraq^21^ reported it to be a significant attribute of ever undergoing screening for cervical cancer. This was concurrent with our observations. Usually, a trained gynaecologist counsels or suggests their patients regarding desirable SRH related practices. On the other hand, those who were more bothered about their own SRH might have more sought periodic gynaecologist consultation.

In our study, only 3.0% of the study subjects had received vaccination for cervical cancer which was quite low. This was not at all surprising as HPV vaccine is not included in India’s national immunisation schedule. One who wishes to take HPV vaccine needs to bear the vaccination cost. Moreover, it is not cheap either. Single dose of HPV costs around 34 to 68 USD in the country. Three doses of HPV are recommended for adequate protection. Out of pocket expenditure is a known barrier of vaccine hesitancy.^22^

In our study, lower age emerged as a significant attribute of cervical cancer vaccine acceptance. This was in line with the existing recommendations for HPV vaccine.^4,6-8^ Although we found that the median age of vaccination of our study subjects to be 30 years. At this age the vaccine provides very limited protection against the disease therefore seldomly recommended. Positive attitude towards the disease significantly influenced vaccine acceptance of our study subjects. The odds of this association (6.4) was more than double compared to odds of association between positive attitude and screening (3.0). This may be because in India HPV vaccination requires out of pocket expenditure unlike cervical cancer screening which can be availed at any government healthcare facility free of cost. Thus, a higher level of positive attitude was required to facilitate vaccination compared to screening in our study subjects.

In limitations, most of the data were self-reported by the study participants. So, there may be reporting and social desirability related biases. Secondly, there might be possibilities that those who were more digitally literate and aware regarding the disease might have participated more. So, there might be response bias and thus generalisability of the study findings to the other schools in the study area was limited. Thirdly, the study was originally planned to be conducted by face-to-face interview method but due the ongoing global pandemic at the time of data collection the researchers opted for online data collection considering safety and feasibility. Lastly, some other possible attributes of cervical cancer screening and vaccination (for e.g., number of sexual partners, age at first sexual exposure etc.) were knowingly omitted considering sensitivity of reporting all these items in Indian context.

## Conclusion

Acceptance of screening and vaccination for cervical cancer among the study subjects were found to be quite low. They must be repeatedly sensitised by the healthcare providers regarding different aspects of cervical cancer on every given opportunity to improve their practices related to the disease. These sensitizations should include awareness of preventable cancer risk factors, importance of early diagnosis, availability of screening in government health facilities and effective vaccine against the disease. Inclusion of sessions on awareness regarding common preventable diseases such as cervical cancer in school teachers’ induction training may be useful. School teachers being an educated member of the society bears tremendous potential to influence health related practices of their peers, students and women of their own communities. Thus, policymakers should more actively involve school teachers in generating awareness and demand for cervical cancer screening and vaccination in their native communities to curb the disease in the country.

## Acknowledgements

We would like to express our gratitude towards all the study participants and head of the participatory institutions of the Kota block in the study. Without their help and unconditional support, especially during an ongoing global pandemic, the study would not have been possible.

## Funding

We received no funding for this research.

## Competing interests

The authors declare that there is no conflict of interests.

## Ethical approval

Ethical clearance of the Institutional Ethics Committee (IEC) of the Maulana Azad University (Ref. No. - MAUJ/DOPH/IRBL-MPH/18-006 dated February 3, 2020) was taken before conducting the research.

## Authors’ contributions

AS conceived and designed the study, conducted research, provided research materials, and collected and organized data. BB helped in the study design, analysed, interpreted data and wrote the initial draft of the manuscript. BS revised and approved the final draft of the article, and provided logistic support. All authors have critically reviewed and approved the final draft and are responsible for the content and similarity index of the manuscript.

**Table 1:** Distribution of the study participants as per their knowledge regarding different aspects of cervical cancer ( n=397)

**Item Number**	**Variable**	**Yes;** **N (%)**	**No;** **N (%)**	**Don’t Know;** **N (%)**
	As far as you are aware, which of the following can or cannot be a risk factor for cervical cancer?			
K-1	Human papilloma virus (HPV) infection	**147 (37.0)**	82 (20.7)	168 (42.3)
K-2	Tobacco consumption and smoking	**174 (43.8)**	163 (41.1)	60 (15.1)
K-3	Lowered immunity (i.e. diabetes, transplant, HIV patients etc.)	**239 (60.2)**	88 (22.2)	70 (17.6)
K-4	Early marriage (before 18 years of age)	**232 (58.4)**	91 (22.9)	74 (18.6)
K-5	Early pregnancy	**254 (64.0)**	79 (19.9)	64 (16.1)
K-6	Multiple pregnancies	**213 (53.7)**	82 (20.7)	102 (25.7)
K-7	Multiple sexual partners	**231 (58.2)**	60 (15.1)	106 (26.7)
K-8	Having sexual partners with many previous partners	**182 (45.8)**	74 (18.6)	141 (35.5)
K-9	Oral contraceptive pill (OCP) intake for long term	**224 (56.4)**	76 (19.1)	97 (24.4)
K-10	Infection with Chlamydia (a sexually transmitted disease)	**186 (46.9)**	54 (13.6)	157 (39.5)
K-11	Poor menstrual hygiene	**333 (83.9)**	28 (7.1)	36 (9.1)
K-12	Not undergoing regular diagnostic check-ups	**215 (54.2)**	106 (26.7)	76 (19.1)
K-13	>Family history of cervical cancer	**231 (58.2)**	104 (26.2)	62 (15.6)
K-14	Higher age	**138 (34.8**	127 (32.0)	132 (33.2)
	As per your knowledge, which of the following can or cannot be sign or symptom of cervical cancer?			
K-15	Menstrual abnormality	**288 (72.5)**	50 (12.6)	59 (14.9)
K-16	Postmenstrual bleeding	**268 (67.5)**	38 (9.6)	91 (22.9)
K-17	Vaginal discharge with a foul smell	**266 (67.0)**	42 (10.6)	89 (22.4)
K-18	Persistent pain in back or legs	**201 (50.6)**	62 (15.6)	134 (33.8)
K-19	Blood in urine or stool	**183 (46.1)**	92 (23.2)	122 (30.7)
K-20	Urinary urgency	**104 (26.2)**	94 (23.7)	199 (50.1)
K-21	Diarrhoea	**24 (6.0)**	218 (54.9)	155 (39.0)
K-22	Pain during sexual intercourse	**204 (51.4)**	46 (11.6)	147 (37.0)
K-23	Bleeding during sexual intercourse	**216 (54.4)**	32 (8.1)	149 (37.5)
K-24	Weight loss	**181 (45.6)**	62 (15.6)	154 (38.8)
	Have you heard about following tests for screening of cervical cancer?			
K-25	Visual inspection under ascetic acid (VIA)	**46 (11.6)**	351 (88.4)	-
K-26	Visual inspection under Lugol’s iodine (VILI)	**32 (8.1)**	365 (91.9)	-
K-27	Pap smear test	**147 (37.0)**	250 (63.0)	-
K-28	If you heard about Pap test, when should one start getting these tests done?			
	After onset of sexual activity	38 (9.6)	-	-
	At 21 years	18 (4.5)	-	-
	At 30 years	**68 (17.1)**	-	-
	Don’t K-now	273 (68.8)	-	-
K-29	At what time interval, Pap test should be repeated for cervical cancer screening?			
	One year after the previous test	68 (17.1)	-	-
	Three years after the previous test	**59 (14.9)**	-	-
	Five years after the previous test	**4 (1.0)**	-	-
	Don’t know	266 (67.0)	-	-
K-30	As per your knowledge, is there any vaccine available to protect against cervical cancer?	**129 (32.5)**	98 (24.7)	170 (42.8)
K-31	What should be the ideal age of receiving vaccine to protect against cervical cancer for a girl or women?			
	9-14 years	**36 (9.1)**	-	-
	Above 15 years	54 (13.6)	-	-
	Anytime during her reproductive life	8 (2.0)	-	-
	Before marriage or onset of sexual activity	31 (7.8)	-	-
	Don’t Know	268 (67.5)	-	-

*Note*. Correct responses are being bolded; K: knowledge; Pap: Papanicolaou.

**Table 2:** Distribution of the study participants as per their attitude regarding cervical cancer (n=397)

**Item Number**	**Variable**	**Agree; N (%)**	**Disagree; N (%)**	**Cannot Say; N (%)**
A-1	Cervical cancer is mostly preventable	**307 (77.3)**	20 (5.0)	70 (17.6)
A-2	Any woman including you can acquire cervical cancer	**319 (80.4)**	18 (4.5)	60 (15.1)
A-3	One should maintain distance from a person who has cervical cancer as it is contagious.	24 (6.0)	**297 (74.8)**	76 (19.1)
A-4	Women should get an internal examination done by a gynaecologist at least once in 3 years to avert the risk of cervical cancer.	**308 (77.6)**	24 (6.0)	65 (16.4)
A-5	One should undergo cervical cancer screening if offered.	**315 (79.3)**	32 (8.1)	50 (12.6)
A-6	A girl should get herself vaccinated before the onset of sexual activity in her life.	**164 (41.3)**	80 (20.2)	153 (38.5)

*Note*. Correct responses are being bolded

**Table 3:** Distribution of the school teachers as per their background characteristics and screening and vaccination status for cervical cancer (n=397)

**Variable**	**N (%)**	**Undergone screening for cervical cancer**	***P*** ** value** ^*^	**Taken vaccine against cervical cancer**	***P*** ** value** ^*^
Age (year)					
≤40 (median)	214 (53.9)	6 (2.8)	0.000	10 (4.7)	0.038
>40	183 (46.1)	27 (14.8)		2 (1.1)	
Educational level					
Undergraduate	116 (29.2)	8 (6.9)	.511	2 (1.7)	0.332
Postgraduate	281 (70.8)	25 (8.9)		10 (3.6)	
Religion					
Hindu	352 (88.7)	24 (6.8)	0.003	12 (3.4)	-
Others^#^	45 (11.3)	9 (20.0)		0 (0.0)	
Ethnicity					
SC/ST/OBC	80 (20.2)	2 (2.5)	0.035	4 (5.0)	0.248
Others	317 (79.8)	31 (9.8)		8 (2.5)	
Marital status					
Unmarried	36 (9.1)	0 (0.0)	-	0 (0.0)	-
Married	338 (85.1)	28 (8.3)		12 (3.6)	
Others	23 (5.8)	5 (21.7)		0 (0.0)	
Had children					
No	57 (14.4)	3 (5.3)	0.368	0 (0.0)	-
Yes	340 (85.6)	30 (8.8)		12 (3.5)	
Type of family					
Nuclear	229 (57.7)	19 (8.3)	0.990	6 (2.6)	0.584
Joint	168 (42.3)	14 (8.3)		6 (3.6)	
Family History of cancer					
No	326 (82.1)	24 (7.4)	0.142	10 (3.1)	0.911
Yes	71 (17.9)	9 (12.7)		2 (2.8)	
Predominantly seek healthcare from					
Government	82 (20.7)	4 (4.3)	0.103	4 (4.3)	0.424
Private	12 (3.0)	29 (9.6)		8 (3.3)	
Per capita monthly family income in USD					
<204 (median)	100 (25.2)	16 (8.2)	0.915	2 (1.0)	0.021
≥204	297 (74.8)	17 (8.5)		10 (5.0)	
Had adequate knowledge regarding the risk factors of the disease: (Yes)	206 (48.1)	22 (10.7)	0.076	8 (3.9)	0.298
Had adequate knowledge regarding the disease sign or symptoms: (Yes)	174 (43.8)	22 (12.6)	0.006	6 (3.4)	0.662
Knew at least one method for cervical cancer screening: (Yes)	173 (43.6)	33 (19.1)	-	10 (5.8)	0.005
Knew ideal age for initiation of cervical cancer screening: (Yes)	86 (21.7)	8 (9.3)	0.707	4 (4.7)	0.319
Had correct knowledge regarding frequency of screening for cervical cancer: (Yes)	63 (15.9)	17 (27.0)	0.000	4 (6.3)	0.093
Knows regarding vaccine availability to protect against cervical cancer: (Yes)	129 (32.5)	23 (17.8)	0.000	12 (9.3)	-
Knew ideal age of vaccination against cervical cancer: (Yes)	36 (9.1)	8 (22.2)	0.002	6 (16.7)	0.000
Had positive attitude towards cervical cancer: (Yes)	114 (28.7)	20 (17.5)	0.003	10 (8.8)	0.000
Used to seek gynaecologist consultation:					
As per need	344 (86.6)	24 (7.0)	0.014	8 (2.3)	0.039
Periodic	53 (13.4)	9 (17.0)		4 (7.5)	

SC: scheduled caste; ST: scheduled tribe; OBC: other backward class; USD: United States dollar. ^*^Chi-square test; ^#^includes 16 Muslim,12 Sikh, 11 Christian and 6 others.

**Table 4:** Univariate and multivariable logistic regression analysis showing strength of association between school teachers screening and vaccination status for cervical cancer with their various attributes (n=397)

**Variable**	**Undergone screening for** **cervical cancer**		**Taken vaccine against** **cervical cancer**	
	**COR (CI)**	**AOR (CI)**	**COR (CI)**	**AOR (CI)**
Age (year)				
≤40 (median)	Ref.	Ref.	4.4 (0.9-20.5)	7.6 (1.5-38.9)
>40	6.0 (2.4-14.9)	5.7 (2.0-15.8)	Ref.	Ref.
Religion				
Hindu	Ref.	Ref.	-	-
Others^*^	3.4 (1.5-7.9)	2.8 (0.9-7.7)		
Ethnicity				
SC/ST/OBC	Ref.	Ref.	-	-
Others	4.2 (0.9-18.0)	2.3 (0.5-11.1)		
Per capita monthly family income in USD				
<204 (median)	-	-	Ref.	Ref.
≥204			5.1 (1.1-23.5)	4.9 (0.9-25.9)
Had adequate knowledge regarding the disease sign or symptoms: (Yes)	2.8 (1.3-5.9)	1.9 (0.7-4.8)	-	-
Knew at least one method for cervical cancer screening: (Yes)	-	-	6.8 (1.5-31.4)	1.9 (0.3-12.8)
Had correct knowledge regarding frequency of screening for cervical cancer: (Yes)	7.3 (3.5-15.5)	6.0 (2.4-15.5)	-	-
Knows regarding vaccine availability to protect against cervical cancer: (Yes)	5.6 (2.6-12.2)	1.3 (0.4-3.8)	-	-
Knew ideal age of vaccination against cervical cancer: (Yes)	3.8 (1.6-9.3)	1.3 0.4-4.3)	11.8 (3.6-38.9)	3.4 (0.7-15.3)
Had positive attitude towards cervical cancer: (Yes)	4.4 (2.1-9.2)	3.0 (1.1-8.0)	13.5 (2.9-62.7)	6.4 (1.1-38.2)
Used to seek gynaecologist consultation:				
As per need	Ref.	Ref.	Ref.	Ref.
Periodic	2.7 (1.2-6.2)	1.4 (1.1-8.6)	3.4 (0.9-11.8)	1.8 (0.4-8.3)
Nagelkerke R^2^	-	.355	-	0.352
Hosmer Lemeshow test p-value	-	.165	-	0.504
Predictive accuracy rate	-	92.7	-	96.0

COR: crude odds ratio; CI: confidence interval; AOR: adjusted odds ratio; SC: scheduled caste; ST: scheduled tribe; OBC: other backward class; USD: United States dollar. ^*^Includes 16 Muslim,12 Sikh, 11 Christian and 6 others.
